# A Retrospective Study of the Adverse Events Associated With Over-the-Counter Hearing Aids in the Manufacturer and User Facility Device Experience (MAUDE) Database

**DOI:** 10.7759/cureus.72491

**Published:** 2024-10-27

**Authors:** Quan Lu, Anita Jeyakumar

**Affiliations:** 1 Otolaryngology, Northeast Ohio Medical University, Rootstown, USA; 2 Otolaryngology, HEARS, LLC, Akron, USA

**Keywords:** adverse events, hearing aid, maude database, non-prescription, over-the-counter

## Abstract

Objective: To examine the adverse events reported in the United States Food and Drug Administration (USFDA)'s Manufacturer and User Facility Device Experience (MAUDE) database for over-the-counter (OTC) or non-prescription hearing aids.

Methods: A retrospective cross-sectional study was performed using the USFDA’s MAUDE database from January 2014 to August 2024. Adverse events were identified using the product codes (QUF, QUG, and QUH) and the keywords (“Hearing Aid, Air-Conduction with Wireless Technology, Over the Counter, Hearing Aid, Air-Conduction, Over the Counter, and Self-Fitting Air-Conduction Hearing Aid, Over the Counter”). Exclusion criteria included reports unrelated to OTC hearing aids and the usage of the device, or those providing insufficient information. The incidence of adverse events was estimated using data from market research reports.

Results: A total of 25 adverse events were identified, with 17 reports meeting the inclusion criteria. Medical device reports (MDRs) were available for 2022 (n=1), 2023 (n=9), and 2024 (n=7). Eighteen (60.0%) reports were related to device malfunction, six (20.0%) were regarding poor customer service, three (10.0%) referred to medical adverse events, two (6.7%) were associated with failure to provide hearing benefits, and one (3.3%) referred to incompatibility with other medical equipment. Nine (30.0%) of the device malfunction reports were due to poor construction or the device falling apart, with eight (88.9%) of those cases resulting in foreign bodies in the ear. The incidence rate of adverse events for OTC hearing aids was calculated as 0.00132%.

Conclusions: There is a low reported incidence of adverse events associated with OTC hearing aids. Possible poor construction of the device and its falling apart, resulting in foreign bodies in the ear, and poor customer service were commonly reported as adverse events. This information can aid providers in advising patients and managing expectations. However, more robust studies are needed to monitor adverse events associated with OTC hearing aids.

## Introduction

It is estimated that between 13 and 23 percent of Americans over 12 years of age are impacted by hearing loss [1,2]. Specifically, over 53 million people experience mild-to-moderate hearing loss in at least one ear, and about 36 million experience mild-to-moderate hearing loss bilaterally [1]. According to the National Institutes of Health (NIH), an estimated 28.8 million Americans could benefit from hearing aid usage, but only between 16 and 30 percent of those individuals have ever used them [2]. Many cited costs as the major barrier to accessing hearing healthcare, with Medicare and private insurance not covering or partially covering the cost of hearing aids. It is estimated that a pair of hearing aids can cost between USD 2000 and 7000 [3,4]. A study has found that an average cost of USD 2500 for hearing aids posed an unaffordable expense for 77% of Americans needing them [5]. However, with the passage of the Food Drug and Administration (FDA) Reauthorization Act and the Over-the-Counter Hearing Aid Act of 2017, which allowed for the creation of a category of hearing aids that can be sold over-the-counter (OTC) without a prescription, an individual seeking hearing aids is no longer required to obtain an evaluation and fitting from a licensed healthcare professional, including an otolaryngologist, audiologist, or authorized dispenser. From October 2022 onwards, these OTC hearing aids have become available for purchase by the United States public at a significantly lower price. This offered a potential solution for individuals aged 18 years and older suffering from mild-to-moderate hearing loss [6,7]. 

The FDA Manufacturer and User Facility Device Experience (MAUDE) database provides a collection of medical device reports (MDRs) for the last ten years from mandatory and voluntary reporters, including manufacturers, importers, device user facilities, healthcare providers, patients, and consumers. To date, no study has analyzed the MDRs from the FDA’s MAUDE database for non-prescription hearing aids. The goal of this study is to characterize and describe the adverse events attributed to OTC hearing aids since they became widely available for purchase. We aim to fill this knowledge gap and provide healthcare providers with information regarding the adverse events associated with non-prescription hearing aids to assist in patient counseling and highlight the need for further research in this area.

This article was accepted as a poster presentation at the 2024 Northeast Ohio Medical University (NEOMED) Student Research Symposium held on November 22, 2024.

## Materials and methods

The study was exempted by the institutional review board at Northeast Ohio Medical University (Protocol Number 24-013). Data from the MAUDE database are de-identified and publicly available. The MAUDE database was queried by two independent investigators for all reports between January 2014 and August 2024 using product codes (QUF, QUG, and QUH) and keywords (“Hearing Aid, Air-Conduction with Wireless Technology, Over The Counter, Hearing Aid, Air-Conduction, Over The Counter, and Self-Fitting Air-Conduction Hearing Aid, Over The Counter”) search in product class to identify medical device reports (MDRs) relating to OTC hearing aids. Reports were then screened for duplication and assessed for suitability for the review. Exclusion criteria included reports unrelated to OTC hearing aids and the usage of the device, and those providing insufficient information. Information abstracted from the MDRs consisted of the device manufacturer, year of event occurrence, and type of event. Discrepancies were resolved through discussions between the two independent reviewers. Results were then categorized into device malfunction, failure to provide hearing benefits, customer service-related issues, medical adverse events, and incompatibility with other devices. MDRs with multiple events were delineated into the appropriate categories. Descriptive data analysis was performed using Microsoft Excel (Microsoft Corp., Redmond, WA). The total number of OTC hearing aid units sold was estimated by obtaining an average of the data from the Grand View Research’s U.S. OTC Hearing Aids Market Size, Share & Trends Analysis Report, 2024-2030 [8] and the Global Market Insights’ U.S. OTC Hearing Aid Market Forecast, 2024-2030 [9]. This data was also used in the estimation of the incidence of adverse events. Based on the average market values and estimated annual growth rates of OTC hearing aids in 2023 obtained from these reports, we estimated the market values in 2022 and up to the third quarter of 2024. We used the estimated average cost of one pair of OTC hearing aids from the reports and calculated the number of pairs and total individual units of OTC hearing aids sold. The incidence rate was calculated by dividing the number of adverse events by the total number of OTC hearing aid units sold and presented as a percentage (the rate per 100,000 units). 

## Results

A total of 25 adverse events were identified from the search strategies, with 17 reports included in the final review. Five duplicate reports were excluded, and three were removed because of exclusion criteria. One of the excluded reports was of a patient being sold an OTC hearing aid for unilateral hearing loss, one of the contraindications of this device as per the FDA, and the other two were due to insufficient information and being unrelated to OTC hearing aids. MDRs were available in 2022 (n=1), 2023 (n=9), and 2024 (n=7). Manufacturers with the highest number of event reports included Nano Hearing Aids® (n=4), Sound Quest, LLC (n=3), and Audien Hearing (n=2). The remaining manufacturers, including Linner, GN Consumer Hearing Corporation, Xiamen Retone Hearing Technology Co. Ltd., Olive Union Inc., iHear Direct, MD Hearing, Huizhou Jinghao Medical Technology Co., Ltd., and hearX Group, had one report each. 

**Figure 1:**
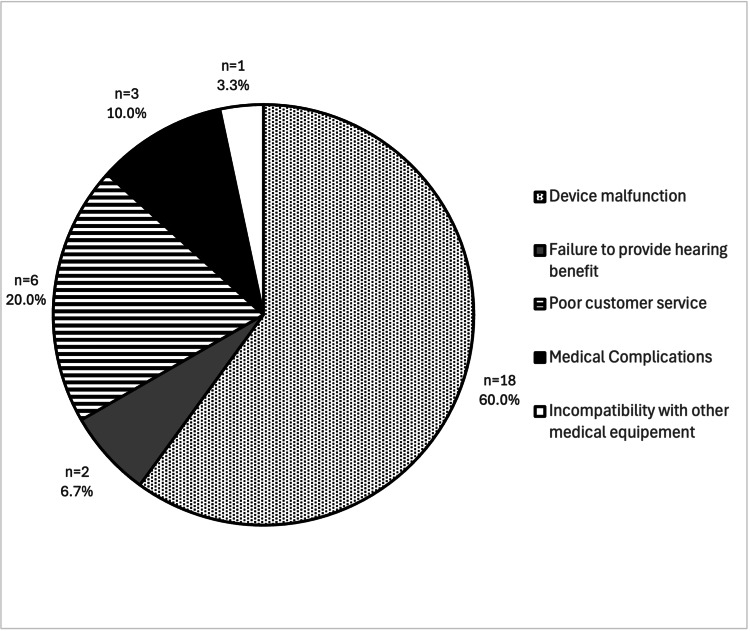
Adverse events of over-the-counter hearing aids as reported in the Manufacturer and User Facility Device Experience (MAUDE) database

Eighteen (60.0%) of the adverse event reports were related to device malfunction, six (20.0%) to poor customer service, three (10.0%) to medical adverse events, two (6.7%) to failure to provide hearing benefits, and one (3.3%) to incompatibility with other medical equipment (Figure 1).

Nine (30.0%) of the device malfunction reports were due to poor construction or the device falling apart, with eight (88.9%) of those cases resulting in a foreign body in the ear. The miscellaneous category included two reports of hearing aid feedback noises and two incidents of volume adjustment issues. Both reports for failure to provide hearing benefits were for the Nano Hearing Aids® Nano CIC Rechargeable devices. Under the category of poor customer service, there were three reports of poor communication or inability to establish communication with the company and three incidents of patients attempting to return the hearing aids and requesting monetary refunds after unsatisfactory trials but only being offered replacements by the company. There was one case report each of the patient contacting the company for support but receiving a request for their credit card information and another patient’s daughter attempting to get a replacement for a non-functional hearing aid found in an unopened box, but being requested additional payment by the company. The medical adverse events delineated consisted of crusty ear lesion (n=1), pruritic ear (n=1), and worsening of migraine (n=1). The one report of incompatibility with other medical devices was in a patient who had a burning sensation in their hip and spine while using a Philips continuous positive airway pressure (CPAP) machine and InterStim implants for bowel incontinence along with the OTC hearing aid. The sensation resolved when the patient removed the hearing aid (Table 1).

**Table 1:** Adverse events of over-the-counter hearing aids by product codes HA: hearing aid, Tech: technology

Event Type	HA with Wireless Tech. (QUG)	HA without Wireless Tech. (QUF)	Self-fitting HA (QUH)	Total Count	% Total
Device malfunction	-	-	-	18	60.0
Poor construction/fell apart	3	5	1	9	30.0
-> Foreign body	2	5	1	8	26.7
-> No foreign body	1	0	0	1	3.3
Charging/battery issue	1	1	0	2	6.7
Nonfunctional without explanation	0	3	0	3	10.0
Miscellaneous	-	-	-	4	13.3
Volume adjustment	0	2	0	2	6.7
Feedback noise	0	1	1	2	6.7
Failure to provide hearing benefit	0	2	0	2	6.7
Poor customer service	2	4	0	6	20.0
Medical adverse events	-	-	-	3	10.0
Skin concerns (irritation, pruritus, etc.)	0	1	1	2	6.7
Exacerbation of pre-existing condition (migraine)	0	1	0	1	3.3
Incompatibility with other medical equipment	0	1	0	1	3.3

As per the Grand View Research Report and Global Market Insights Report, in 2023, the US OTC hearing aid market was estimated at USD 586.1 million and USD 123 million, respectively, producing an average market value of USD 354.55 million. Given that the OTC hearing aid market is estimated to grow every year at a compound annual growth rate of between 5.8 and 9.0% (an average rate of 7.4%), we estimated that it would have been worth USD 328.313 million in 2022 and USD 285.59 million up to the third quarter of 2024. We then estimated that the total pairs of OTC hearing aids sold in the above period were 645,635 pairs based on the estimated average cost of a pair of OTC hearing aids (USD 1,500). Based on the rough estimate of 1.291 million total units sold between 2022 and the third quarter of 2024 and 17 MDRs of adverse events in the MAUDE database, the incidence rate of adverse events is approximately 1.32 per 100,000 units or 0.00132%.

## Discussion

In the current study, we offered a preliminary look at the types and frequency of adverse events reported for OTC hearing aids two years after they became available. As with all newly introduced devices, there are concerns regarding the safety profile of OTC hearing aids, with 75.9% of hearing healthcare providers reporting this concern [10]. Our findings differed from the current literature on complications related to OTC hearing aid usage in that the most reported event was device malfunction in the form of disconnection of the hearing aid components resulting in foreign bodies in the ear. However, the FDA and the OTC hearing aid user manuals warn of foreign bodies, along with skin irritation, abrasions or burns from the device, and sudden worsening of hearing loss as possible adverse effects [6,11]. This deviation could reflect real-world device usage, where users are influenced by extraneous factors in daily life, resulting in improper handling of the devices. However, the medical adverse events identified by our study such as crusty ear lesions, ear pruritus, and exacerbation of migraine have also been mentioned in other studies. Conversely, some literature also reported no adverse effects related to OTC hearing aid usage. The single-center open-label study by Sacco et al. involving 31 patients observed no adverse event [12]. A randomized clinical trial by De Sousa et al. included patients using OTC devices (n=32) and audiologist-fitted devices (n=32) and found only one report of a middle ear infection related to the former and no adverse events in the latter group [13]. A follow-up study monitoring patients eight months after receiving OTC or audiologist-fitted hearing aids reported that one patient with an OTC device required assistance with intermittent static, and one patient with an audiologist-fitted device needed support with gain adjustments [14]. Similarly, the FDA publication of the Jabra Enhance Plus hearing aid Substantial Equivalence Determination reported no adverse events in the trials with those hearing aids [15]. However, a study in France conducted on patients using the Sonalto hearing aid (n=47), a pre-set OTC hearing aid, reported 13 events (all classified as mild). The study observed eight cases of ear pruritus, three cases of auricular pain, one case of external ear inflammation, and another one of external otitis. Only the case of otitis externa required discontinuation of the hearing aid [16]. To place these adverse events in the context of hearing aids as a broad category of medical devices, studies have found that commonly reported side effects of fitted and prescription hearing aids included sounds being too soft or uncomfortably loud, not picking up people speaking in noisy/windy environments and certain voices, poor sound quality, wax build-up, itching and rashes, feedback noise, and tinnitus [17,18]. In addition, a recent study found that OTC hearing aids were comparable to prescription hearing aids in safety when assessing the risk of noise-induced sensorineural hearing loss from overamplification [19].

Though we calculated the incidence rate of adverse events for OTC hearing aids as approximately 1.32 per 100,000 units or 0.00132% based on the Grand View Research’s and Global Market Insights’ OTC Hearing Aids Market Size, Share & Trends Analysis Reports, the true incidence and prevalence remain unclear. According to the Hearing Industries Association (HIA), the sale of OTC hearing aids is estimated to be between 100,000 and one million units with no organization tracking sales, preventing accurate accounting of the numbers being sold since their approval [20]. The variation in the OTC hearing aid market size was also reflected in the drastically different estimated values provided by the two reports. Incidence rate calculation was also complicated by underreporting and incomplete reporting of adverse events to the FDA. Contributing factors could include OTC hearing aid users who may not associate them with medical devices or are unaware of the option to report adverse events to the FDA without support and guidance from hearing healthcare providers. 

Removing the prescription requirement for hearing aids has reduced some of the barriers to accessing hearing healthcare. However, it also highlights the disadvantages of potentially decreased patient education and awareness, as well as insufficient user support, and can exacerbate the disparity in those with low health and digital literacy. The concern about the lack of adequate servicing was acknowledged by 81.4% of hearing healthcare providers [10]. Our study found that 20% of the reported events were related to poor customer service, especially when the user encounters an issue and requires support, exchange, or refund. This finding highlights the vital role of customer support, particularly for older adults, as demonstrated by the case report from Berenbrok et al., where the patients depended on the pharmacists for setup and troubleshooting when they ran into a device malfunction [21]. The data showed that between 34.48 and 87.50% of hearing aid users had not sought help for a problem with their device, and between 12.5 and 65.45% of users reported a problem. Yet, the problem persists, further substantiating the need for companies in the OTC hearing space to bolster their customer relations and support system [17]. This will ensure that individuals attempting to access hearing healthcare can feel confident in their decision and can truly benefit from the increased accessibility brought forth by this new category of hearing aids. Another concern raised by 89.6% of hearing healthcare providers was missing medical red flags [10]. Only one such incident occurred in our study during the review period when a patient was sold an OTC hearing aid for unilateral hearing loss. Other “red flag” conditions necessitating physician visits include birth defects, ear trauma, blood or drainage from the ear, otalgia, excess cerumen or foreign body in the ear, vertigo, sudden hearing changes, and unilateral tinnitus [6]. In addition, 66% of patients were slightly to extremely uncomfortable with purchasing a hearing aid online without a hearing test (of note, the OTC hearing aids are FDA-approved for mild-to-moderate hearing loss) [22]. These findings indicate a need for continued public education regarding the comparable effectiveness of OTC and prescription hearing aids [14], the targeted population that would most benefit from these devices, realistic expectations of OTC hearing aid usage, and when to seek hearing healthcare providers to escalate care.

We had one report of a possible interaction between the OTC hearing aid, the InterStim neurostimulation system for bowel control, and the CPAP machine. A review of the Medtronic user information identified possible interactions with cardiac devices, electrocautery, defibrillators, ultrasonic equipment, radiation therapy, MRI, and theft detectors/screening devices. Adverse events for the InterStim system included pain at implant sites, new pain, lead migration, infection, technical or device problems, adverse change in bowel or voiding function, and undesirable stimulation or sensations, including jolting or shock sensations [23]. It was unclear from the MDR whether the devices were conclusively linked to the adverse interaction. 

Limitations of the study included underreporting of adverse events and insufficient data on psychological and social effects of OTC hearing aids reported in the MAUDE database, including the common concerns of fear of missing out on conversation, fear of losing or destroying hearing aids, people talking louder, and less respect from others. In addition, the true incidence and prevalence of adverse events remained unknown due to incomplete information and underreporting from voluntary reporters. The database also suffers from incomplete and imprecise reports that can be difficult to verify. However, our study provides an overview of the adverse effects and user complaints against OTC hearing aids that can aid in provider/patient education and user expectation management, and inspire more robust studies in the future to better understand the safety profile of OTC hearing aids. 

## Conclusions

There is a low reported incidence of adverse events for OTC hearing aids. Potential poor construction, resulting in foreign bodies in the ear, and poor customer service were the most commonly reported adverse events about the OTC hearing aids. Medical adverse events reported consisted of crusty ear lesions, pruritic ear, and exacerbation of migraine, which may or may not have been related to the hearing aids. The data and information regarding adverse events continue to require further development to aid providers in advising existing and prospective patients on OTC hearing aids and managing expectations around their benefits and limitations.
